# Reasons for Downgrading the Certainty of Evidence for Indirectness in Synthesis of Surgical Procedures for Patients With Fractures: A Meta‐Research Analysis

**DOI:** 10.1111/jep.70091

**Published:** 2025-04-06

**Authors:** Julia Pozzetti Daou, Rachel Riera, Rafael Leite Pacheco

**Affiliations:** ^1^ Escola Paulista de Medicina Universidade Federal de São Paulo (Unifesp) São Paulo Brazil; ^2^ Hospital Sírio‐Libanês (HSL) São Paulo Brazil; ^3^ Centro Universitário São Camilo (CUSC) São Paulo Brazil

**Keywords:** bone fractures, GRADE approach, systematic reviews as topic

## Abstract

**Rationale:**

Indirectness occurs when the synthesized evidence may not be directly applied to the relevant clinical context. A common argument used by surgeons is that evidence that arises from clinical trials is limited due to a lack of fidelity regarding surgery techniques, materials, and surgeon and center experience. Considering that there are many particularities in surgical interventions for the treatment of bone fractures, diverseness is expected among trials that randomized patients to compare surgical procedures. An in‐depth analysis of how this expected diverseness is reflected in indirectness judgments on the certainty of synthesized evidence is lacking.

**Aims and Objectives:**

To analyze the certainty of evidence from all Cochrane reviews of surgical interventions for the treatment of patients with bone fractures and the reasons for indirectness downgrading.

**Method:**

A meta‐research analysis of all Cochrane systematic reviews that compare any surgical interventions in patients with any type of bone fracture. A sensitive search was conducted in the Cochrane Database of Systematic Review from inception to 16 October 2024.

**Results:**

The certainty of the evidence of all Cochrane reviews of surgical interventions for patients with fractures is very low or low in 66.5% of eligible outcomes. Indirectness contributed to the certainty downgrade in only 12.26% of outcomes (26/212), and of those, the indirectness was related to the intervention in 11.5% (4/26). The results show that although the certainty of evidence of surgical interventions for patients with fractures is usually downgraded, indirectness is not a common cause of concern.

**Conclusion:**

The certainty of evidence for surgical interventions in patients with fractures is typically downgraded, often to very low. However, indirectness is not a common reason for such downgrading. The anticipated diverseness regarding surgery techniques, materials, and surgeon and center experience was not impactful in the overall certainty of evidence in the Cochrane reviews that were included.

## 1 Introduction

The GRADE approach proposes a framework to assess the certainty of the evidence and to make clinical practice recommendations [1]. To GRADE the certainty of the body of the evidence, five domains of assessment are considered to downgrade the certainty in each outcome estimate: imprecision, inconsistency, indirectness, publication bias, and risk of bias (limitations in study design or execution) [2].

Indirectness arises when the synthesized evidence is not directly applied exactly to the research question. In the words of the GRADE working group, ‘Direct evidence comes from research that directly compares the interventions in which we are interested when applied to the populations in which we are interested and measures outcomes important to patients’ [3].

Three proposed mechanisms of indirectness are related to differences between the intended and studied populations, interventions, and outcomes [3]. A fourth proposed mechanism is related to lack of head‐to‐head trials for the research question when the only available evidence arises from indirect treatment comparisons [3].

In regard to surgical treatment, including those for the treatment of bone fractures, special attention to reasons of indirectness is required. A common argument used by surgeons is that evidence that arises from clinical trials is limited due to a lack of consistency regarding surgery techniques, materials, and surgeon and center experience.

Issues regarding the applicability of the evidence are highlighted in previous GRADE guidance [3]. It is reported that, for instance, interventions may be delivered in different settings or by professionals with varying levels of expertise.

It is important to note that downgrading due to indirectness because of differences in interventions must be carefully considered. Some level of heterogeneity is expected in surgical interventions, and indirectness should only downgrade the certainty of evidence if it is likely sufficient to affect the outcome estimate [3].

Considering that there are many particularities in surgical interventions for the treatment of bone fractures, diverseness is expected among trials that randomized patients to compare surgical procedures. However, an in‐depth analysis of how this expected diverseness is reflected in indirectness judgments on the certainty of synthesized evidence is lacking.

The objective of this study is to analyze the certainty of evidence from all Cochrane reviews of surgical interventions for the treatment of patients with bone fractures and the reasons for indirectness downgrading.

## 2 Methods

### 2.1 Study Design and Setting

A meta‐research analysis was performed at the Discipline of Evidence‐Based Medicine from the Escola Paulista de Medicina, Universidade Federal de São Paulo (Unifesp).

### 2.2 Eligibility Criteria and Unity of Analysis

All Cochrane systematic reviews that compared any surgical interventions in patients with any type of bone fracture were included. From the included Cochrane reviews, all Summary of Findings tables were considered. Finally, each outcome that had the certainty of evidence graded was extracted and analyzed.

### 2.3 Study Identification and Selection Process

A search was conducted on 16 October 2024 in the Cochrane Database of Systematic Review, using the search strategy presented in File S1. The search strategy included the Mesh term for bone fractures and synonyms to increase sensitivity. The list of included reviews is presented in the File S2.

All retrieved references were screened by two independent authors (RLP and JPD) using the Rayyan platform. Any divergence in the inclusion decision was solved by consulting a third researcher (RR).

### 2.4 Data Extraction, Analysis and Presentation

Two independent authors (RLP and JPD) used a standardized extraction sheet to extract relevant data from the included Cochrane reviews. A pilot extraction considering five Cochrane reviews was used to optimize the extraction sheet.

Data analysis was limited to information that was presented by the included Cochrane reviews. The data regarding GRADE judgments was exclusively extracted from the Summary of Findings tables, including the justifications in the footnotes. No reanalysis or analysis of the judgment adequacy was performed.

The certainty of evidence (high, moderate, low, very low) of each eligible outcome from the considered summary of findings table was described. The judgments for downgrading each outcome were also extracted and presented in a bar graph considering the number of eligible outcomes as the denominator.

A sankey plot was also presented correlating the certainty of evidence and GRADE domain, using the user package “sankey” version 1.81 in Stata [4].

The justifications for downgrading for indirectness were quoted in a table, and its content was analyzed. The reason for indirectness was classified by two independent authors (RLP and JPD) in considering the following categories: population‐related, intervention‐related, outcome‐related, data nature (absence of head‐to‐head trials) or not reported.

Data management, statistical analysis, graphs, and tabulation were conducted using Stata version 18. The statistical analysis focused on descriptive measures. Categorical variables were represented as percentages, while numerical variables, due to their skewness, were reported as medians along with the first and third quartiles, minimum values, and maximum values.

## 3 Results

The search strategy retrieved 1101 references. After the selection process, 51 Cochrane reviews were considered eligible and included for analysis. The reviews included had, in total, 39 Summary of Findings tables and 212 eligible outcomes. The main characteristics of the included Cochrane reviews are described in Table 1.

**Table 1:** Main characteristics from the included Cochrane reviews.

Characteristics	
Year of publication	Median = 2016 [IQR = 2013–2020; Range = 1997–2024]
Fracture topography	
Ankle	3.92% (2/51)
Calcaneus	1.96% (1/51)
Distal tibia	1.96% (1/51)
Elbow	7.84% (4/51)
Facial	5.88% (3/51)
Hip	29.41% (15/51)
Knee	5.88% (3/51)
Rib	1.96% (1/51)
Shaft of long bones	11.76% (6/51)
Shoulder	5.88% (3/51)
Stress	1.96% (1/51)
Vertebral	7.84% (4/51)
Wrist	11.76% (6/51)
Nonspecific	1.96% (1/51)
Population	
Children	7.84% (4/51)
Children and adults	5.88% (3/51)
Adults	86.27% (44/51)
Number of summary of findings table	Median = 0 [IQR = 0–1; Range = 0–5]
0	60.78% (31/51)
1	23.53% (12/51)
2	1.96% (1/51)
3	7.84% (4/51)
4	3.92% (2/51)
5	1.96% (1/51)
Total	39 summary of findings table
Number of eligible outcome assessments	Median = 6 [IQR = 5.5–16.5; Range 4–29]
1–10	65% (13/20)*
11–20	20% (4/20)*
21–30	15% (3/20)*
Total	212 eligible outcomes

*Note:* Interquartile Range (IQR) = Quartile (Q)1–Q3, Range = Minimum–Maximum.

* Denominator considers reviews with at least one summary of findings table.

**Figure 1:**
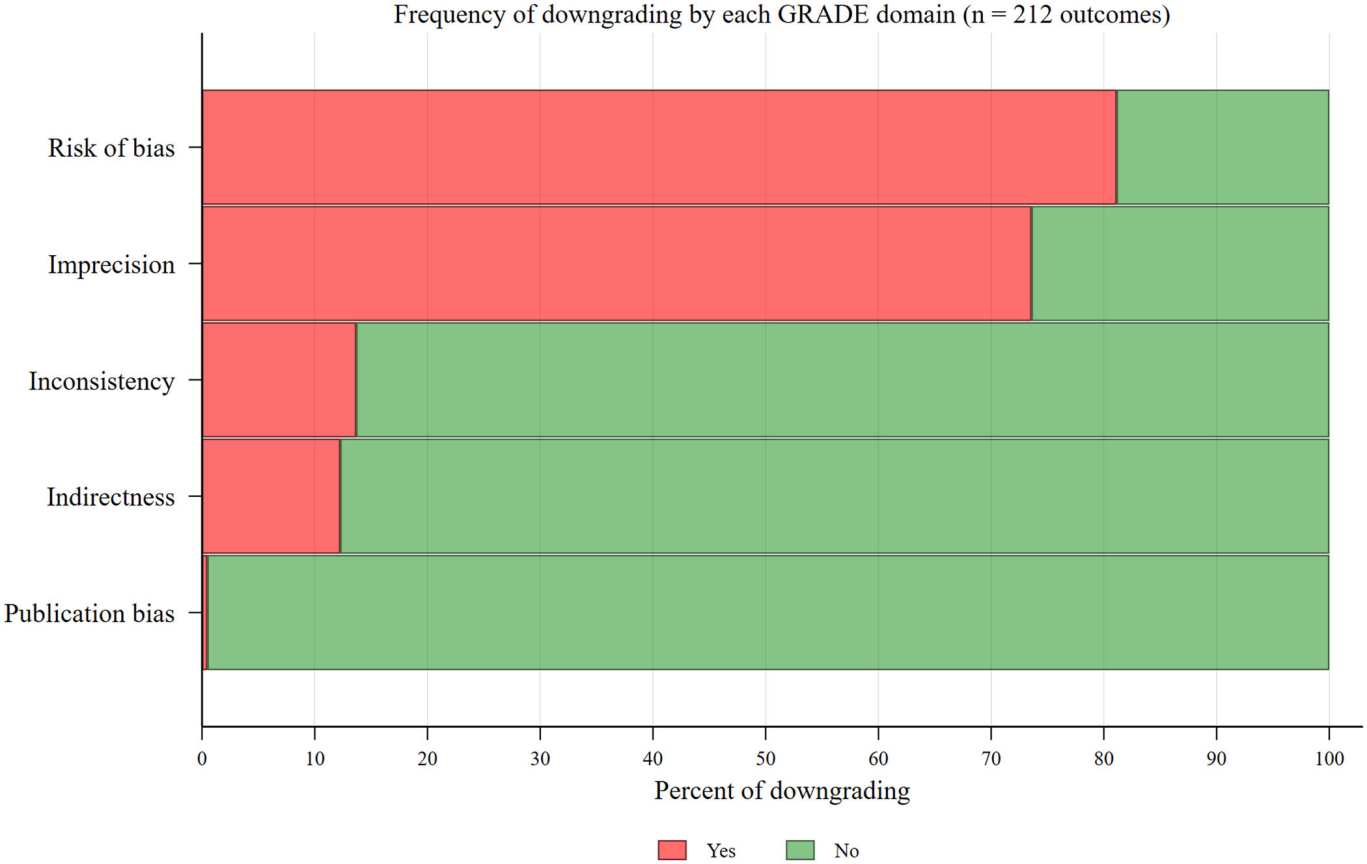
Frequency of domain downgrading in eligible outcomes. [Color figure can be viewed at wileyonlinelibrary.com]

**Figure 2:**
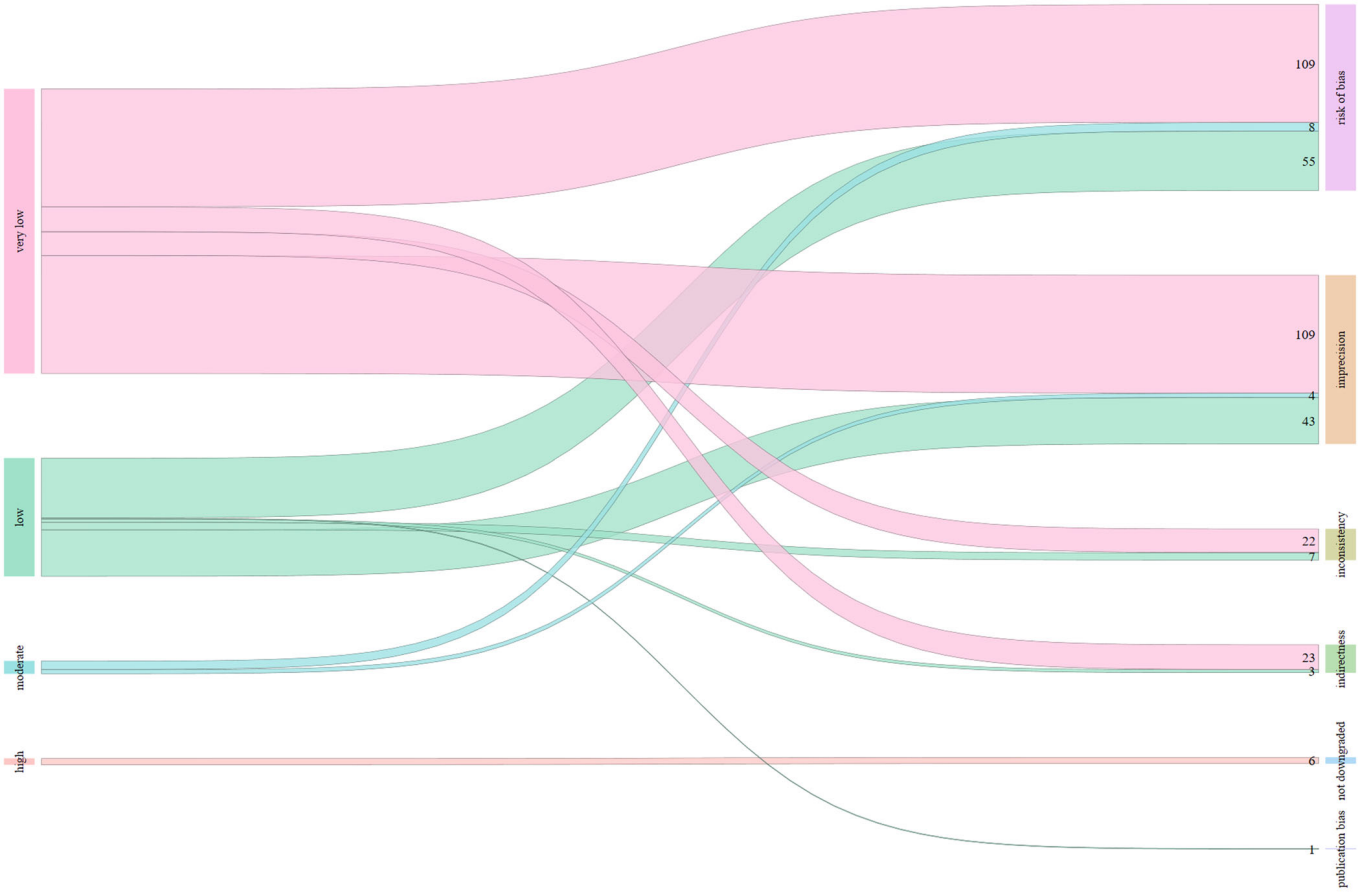
Downgraded domain for each certainty of evidence category. [Color figure can be viewed at wileyonlinelibrary.com]

The certainty of evidence for the 212 eligible outcomes and the reasons for downgrading are presented in Table 2. Figure 1 presents the number of times a GRADE domain was downgraded in each eligible outcome, and Figure 2 presents the downgraded domain for each certainty of evidence category.

**Table 2:** Certainty of evidence characteristics.

Certainty of the evidence	
High	2.83% (6/212)
Moderate	30.66% (65/212)
Low	5.66% (12/212)
Very low	60.85% (129/212)
Downgraded due to	
Risk of bias	81.13% (172/212)
Inconsistency	13.68% (29/212)
Imprecision	73.58% (156/212)
Indirectness	12.26% (26/212)
Publication bias	0.47% (1/212)

As depicted in Figure 1 and Table 2, 26 eligible outcomes (12.26%) were downgraded due to indirectness.

Table 3 depicts the justification quote from the summary of findings table used to downgrade for indirectness. The content of the justification was analyzed, and indirectness was related to the Outcome in 53.8% (14/26); Population in 3.8% (1/26); Population and Outcome in 3.8% (1/26); Population and intervention in 11.5% (4/26); Nature of data in 11.5% (3/26), and not reported in 11.5% (3/26).

**Table 3:** Examples of reasons for downgrading indirectness.

Number of outcomes	Justification quote	Category
**1**	“One level for indirectness (the timing of the outcome was too short considering that the cast was retained for three weeks)”	Outcome
**1**	“One level for indirectness (issues relating to reporting and definition of adverse events)”	Outcome
**1**	“Indirectness (study population included children with sprains only)”	Population
**3**	“Intransitivity: indirect estimates included variation in proportion of stable/unstable fractures and intransitivity may be evident (downgraded by one level)”	Type of data
**1**	“1 level for indirectness (the Herm criteria were not validated and could be considered surrogate to functional outcome)”	Outcome
**1**	“One level for serious indirectness foran incompletely reported outcome measure (see footnote ‘g’)”	Outcome
**1**	“By one level for indirectness, given the measure was poorly defined.”	Outcome
**1**	“By one level for serious indirectness for an inadequately reported outcome measure”	Outcome
**1**	“By one level for serious indirectness as the outcome was not a full measure of satisfaction”	Outcome
**2**	“By one level for indirectness (the scoring system is validated for children with cerebral palsy)”	Outcome
**1**	“By one level for indirectness (as well as variation in the type of fracture, decisions and decision criteria for remanipulation varied)”	Population/Outcome
**1**	“By one level for serious indirectness, reflecting variation or lack of definitions of some complications, including redisplacement”	Outcome
**1**	“By one level for serious indirectness (the evidence was not available for the other potential complications)”	Outcome
**4**	“Downgraded one level for indirectness because of the poorly defined and poor quality outcome measures and for the percutaneous versus open surgery comparison, wherenovel self‐developed techniques were used in the percutaneous surgery group”	Population/Intervention
**2**	“One level for serious indirectness (questions overoutcome measure's validity; the individual importance of the 22 problems covered in the Croft measure will vary)”	Outcome
**1**	“One level for serious indirectness as the definition, measurement and severity of adverse outcomes varied between trials”	Outcome
**3**	Justification not reported	—

## 4 Discussion

The certainty of the evidence of all Cochrane reviews of surgical interventions for patients with fractures is very low or low in 66.5% of eligible outcomes. Indirectness contributed to the certainty downgrade in only 12.26% of outcomes (26/212), and of those, the indirectness was related to the intervention in 11.5% (4/26).

The results show that although the certainty of evidence of surgical interventions for patients with fractures is usually downgraded, indirectness is not a common reason for such downgrading. Furthermore, the outcomes that were downgraded by indirectness had an issue regarding the applicability of an outcome measure.

The anticipated heterogeneity between trials regarding surgery techniques, materials, and surgeon and center experience was not impactful in the overall certainty of evidence in the included Cochrane reviews. Two hypotheses can be drawn from this data. There is not enough heterogeneity translated into certainty‐threatening indirectness, or the GRADE application is not sensitive enough to detect intervention‐related indirectness.

The data in Figure 2 shows that beyond being an infrequent reason for concern, indirectness was only downgraded in evidence that was already downgraded due to the risk of bias or imprecision. There was not a single case where indirectness was a concern on moderate certainty evidence.

The GRADE approach utilizes different frameworks for assessing the certainty of evidence in systematic reviews and for making recommendations in clinical practice guidelines. In more contextualized scenarios, such as clinical practice guidelines, downgrading judgments due to indirectness may be more frequent, which could partly explain the findings of this analysis.

Other analyses showed that high certainty of evidence from different samples of systematic reviews is uncommon [5, 6]. An analysis from 2016 estimated in a sample of 1,394 Cochrane reviews that the certainty of evidence was high for the first listed primary outcome in 13.5% of reviews [5]. Another similar analysis from 2022 estimated that the certainty of evidence was high for at least one primary outcome in 12% of the 2428 analyzed Cochrane reviews [6].

There is also evidence in the literature that shows that indirectness is a domain that is infrequently downgraded in Cochrane reviews. Still, the analysis did not investigate reasons for indirectness downgrade or the category of indirectness [7, 8].

We could not find other examples in the literature that tried to understand the indirectness effects on the certainty of evidence in a sample of published systematic reviews investigating surgical interventions for patients with fractures.

An analysis restricted to evidence in oral health also showed that the certainty of evidence is usually low or very low (88%) and is not increasing over time [9]. Although a different field, oral health reviews usually consider procedural/surgical interventions. Other investigations also concluded that high‐ GRADE ratings are not present in complex interventions [10].

Thus, it was expected that the risk of bias and imprecision were the most common downgraded domains, but the data from this study shows the paucity of sufficient (precise) evidence from low risk of bias trials that were threatened due nature of interventions.

This study has some limitations. First, it is limited to reviews with surgical interventions for any type of fracture. Almost all information was extracted from the Summary of Findings tables from Cochrane reviews, and no analysis of the completeness and adequacy of the judgments was performed. Lastly, the low number of included outcomes that were downgraded due to indirectness prevented a robust analysis of how intervention heterogeneity affects the certainty of evidence in systematic reviews of surgical interventions.

This article adds to the literature of meta‐research articles that aimed to diagnosis and explore methodological particularities in surgical‐oriented fields [11, 12]. As practical implications of this analysis, trialists and systematic reviews can consider better describing and discussing expected heterogeneity between surgical interventions.

Future research should focus on understanding if indirectness related to heterogeneity in surgical interventions is indeed not a major concern or if the current framework for assessing the certainty of evidence in systematic reviews is not able to incorporate this source of indirectness into its judgments. It is also important to see if these results are consistent in other surgical subspecialities.

## 5 Conclusions

The certainty of evidence for surgical interventions in patients with fractures is typically downgraded, often to very low. However, indirectness is not a common reason for such downgrading. The anticipated diverseness regarding surgery techniques, materials, and surgeon and center experience was not impactful in the overall certainty of evidence in the Cochrane reviews that were included.

## Conflicts of Interest

The authors declare no conflicts of interest.

## Supporting information


**Supporting file 1**. Search strategy at Cochrane Database of Systematic Review on 16 October 2024. **Supporting file 2**. List of included systematic reviews.

## Data Availability

Data is available upon reasonable request.
