# Supplementary material for: Reasons for Downgrading the Certainty of Evidence for Indirectness in Synthesis of Surgical Procedures for Patients With Fractures: A Meta‐Research Analysis
Source: J Eval Clin Pract. 2025 Apr 6;31(3):e70091. doi: 10.1111/jep.70091 (PMC11973413; doi:10.1111/jep.70091)
Supplement: Supplementary file 1 — Supporting file 1. Search strategy at Cochrane Database of Systematic Review on 16 October 2024. Supporting file 2. List of included systematic reviews. [file JEP-31-0-s001.docx]

**Supplementary files**

**Reasons for downgrading the certainty of evidence for indirectness in synthesis of surgical procedures for patients with fractures: a meta-research analysis**

*Julia Pozzetti Daou, Rachel Riera, Rafael Leite Pacheco*

**Supplementary file 1**. Search strategy at Cochrane Database of Systematic Review on 16 October 2024

| #1 MeSH descriptor: [Fractures, Bone] explode all trees  #2 (Fracture) OR (Fractures) OR (Bone Fracture) OR (Bone Fractures) OR (Broken Bones) OR (Broken Bone) OR (Spiral Fractures) OR (Spiral Fracture) OR (Torsion Fractures) OR (Torsion Fracture)  #3 #1 OR #2 |
| --- |

**Supplementary file 2**. List of included systematic reviews

| **Review** | **Title** | **URL** | **Year** |
| --- | --- | --- | --- |
| 1 | Arthroplasties (with and without bone cement) for proximal femoral fractures in adults | http://dx.doi.org/10.1002/14651858.CD001706.pub4 | 2010 |
| 2 | Arthroplasties for hip fracture in adults | http://dx.doi.org/10.1002/14651858.CD013410.pub2 | 2022 |
| 3 | Bone grafts and bone substitutes for treating distal radial fractures in adults | http://dx.doi.org/10.1002/14651858.CD006836.pub2 | 2008 |
| 4 | Bone morphogenetic protein (BMP) for fracture healing in adults | http://dx.doi.org/10.1002/14651858.CD006950.pub2 | 2010 |
| 5 | Cephalomedullary nails versus extramedullary implants for extracapsular hip fractures in older adults | http://dx.doi.org/10.1002/14651858.CD000093.pub6 | 2022 |
| 6 | Condylocephalic nails versus extramedullary implants for extracapsular hip fractures | http://dx.doi.org/10.1002/14651858.CD000338 | 1998 |
| 7 | Conservative versus operative treatment for hip fractures in adults | http://dx.doi.org/10.1002/14651858.CD000337.pub2 | 2008 |
| 8 | Different methods of external fixation for treating distal radial fractures in adults | http://dx.doi.org/10.1002/14651858.CD006522.pub2 | 2008 |
| 9 | Dynamic compression plating versus locked intramedullary nailing for humeral shaft fractures in adults | http://dx.doi.org/10.1002/14651858.CD005959.pub2 | 2011 |
| 10 | External fixation versus conservative treatment for distal radial fractures in adults | http://dx.doi.org/10.1002/14651858.CD006194.pub2 | 2007 |
| 11 | Extramedullary fixation implants and external fixators for extracapsular hip fractures in adults | http://dx.doi.org/10.1002/14651858.CD000339.pub3 | 2013 |
| 12 | Internal fixation implants for intracapsular hip fractures in adults | http://dx.doi.org/10.1002/14651858.CD001467 | 2001 |
| 13 | Internal fixation implants for intracapsular hip fractures in older adults | http://dx.doi.org/10.1002/14651858.CD013409.pub2 | 2021 |
| 14 | Internal fixation versus arthroplasty for intracapsular proximal femoral fractures in adults | http://dx.doi.org/10.1002/14651858.CD001708.pub2 | 2006 |
| 15 | Interventions for the management of mandibular fractures | http://dx.doi.org/10.1002/14651858.CD006087.pub3 | 2013 |
| 16 | Interventions for the treatment of fractures of the mandibular condyle | http://dx.doi.org/10.1002/14651858.CD006538.pub2 | 2010 |
| 17 | Interventions for treating ankle fractures in children | http://dx.doi.org/10.1002/14651858.CD010836.pub2 | 2016 |
| 18 | Interventions for treating femoral shaft fractures in children and adolescents | http://dx.doi.org/10.1002/14651858.CD009076.pub2 | 2014 |
| 19 | Interventions for treating fractures of the distal femur in adults | http://dx.doi.org/10.1002/14651858.CD010606.pub3 | 2022 |
| 20 | Interventions for treating fractures of the patella in adults | http://dx.doi.org/10.1002/14651858.CD009651.pub3 | 2021 |
| 21 | Interventions for treating isolated diaphyseal fractures of the ulna in adults | http://dx.doi.org/10.1002/14651858.CD000523.pub4 | 2012 |
| 22 | Interventions for treating proximal humeral fractures in adults | http://dx.doi.org/10.1002/14651858.CD000434.pub5 | 2022 |
| 23 | Interventions for treating supracondylar elbow fractures in children | http://dx.doi.org/10.1002/14651858.CD013609.pub2 | 2022 |
| 24 | Interventions for treating traumatised permanent front teeth: luxated (dislodged) teeth | http://dx.doi.org/10.1002/14651858.CD006203.pub2 | 2013 |
| 25 | Interventions for treating wrist fractures in children | http://dx.doi.org/10.1002/14651858.CD012470.pub2 | 2018 |
| 26 | Intramedullary nailing for tibial shaft fractures in adults | http://dx.doi.org/10.1002/14651858.CD008241.pub2 | 2012 |
| 27 | Intramedullary nails for extracapsular hip fractures in adults | http://dx.doi.org/10.1002/14651858.CD004961.pub4 | 2014 |
| 28 | Osteotomy, compression and other modifications of surgical techniques for internal fixation of extracapsular hip fractures | http://dx.doi.org/10.1002/14651858.CD000522.pub2 | 2009 |
| 29 | Pedicle screw fixation for traumatic fractures of the thoracic and lumbar spine | http://dx.doi.org/10.1002/14651858.CD009073.pub2 | 2013 |
| 30 | Percutaneous pinning for treating distal radial fractures in adults | http://dx.doi.org/10.1002/14651858.CD006080.pub3 | 2020 |
| 31 | Percutaneous vertebroplasty for osteoporotic vertebral compression fracture | http://dx.doi.org/10.1002/14651858.CD006349.pub4 | 2018 |
| 32 | Replacement arthroplasty versus internal fixation for extracapsular hip fractures in adults | http://dx.doi.org/10.1002/14651858.CD000086.pub2 | 2006 |
| 33 | Resorbable versus titanium plates for facial fractures | http://dx.doi.org/10.1002/14651858.CD007158.pub3 | 2018 |
| 34 | Surgical approaches and ancillary techniques for internal fixation of intracapsular proximal femoral fractures | http://dx.doi.org/10.1002/14651858.CD001705.pub2 | 2005 |
| 35 | Surgical fixation methods for tibial plateau fractures | http://dx.doi.org/10.1002/14651858.CD009679.pub3 | 2024 |
| 36 | Surgical interventions for diaphyseal fractures of the radius and ulna in children | http://dx.doi.org/10.1002/14651858.CD007907.pub2 | 2011 |
| 37 | Surgical interventions for treating acute fractures or non‐union of the middle third of the clavicle | http://dx.doi.org/10.1002/14651858.CD007428.pub3 | 2015 |
| 38 | Surgical interventions for treating distal humeral fractures in adults | http://dx.doi.org/10.1002/14651858.CD009890.pub2 | 2013 |
| 39 | Surgical interventions for treating distal radial fractures in adults | http://dx.doi.org/10.1002/14651858.CD003209.pub2 | 2009 |
| 40 | Surgical interventions for treating distal tibial metaphyseal fractures in adults | http://dx.doi.org/10.1002/14651858.CD010261.pub2 | 2015 |
| 41 | Surgical interventions for treating extracapsular hip fractures in older adults: a network meta‐analysis | http://dx.doi.org/10.1002/14651858.CD013405.pub2 | 2022 |
| 42 | Surgical interventions for treating fractures of the olecranon in adults | http://dx.doi.org/10.1002/14651858.CD010144.pub2 | 2014 |
| 43 | Surgical interventions for treating intracapsular hip fractures in older adults: a network meta‐analysis | http://dx.doi.org/10.1002/14651858.CD013404.pub2 | 2022 |
| 44 | Surgical interventions for treating radial head fractures in adults | http://dx.doi.org/10.1002/14651858.CD008987.pub2 | 2013 |
| 45 | Surgical versus conservative interventions for treating ankle fractures in adults | http://dx.doi.org/10.1002/14651858.CD008470.pub2 | 2012 |
| 46 | Surgical versus conservative interventions for treating fractures of the middle third of the clavicle | http://dx.doi.org/10.1002/14651858.CD009363.pub3 | 2019 |
| 47 | Surgical versus conservative management for odontoid fractures | http://dx.doi.org/10.1002/14651858.CD005078.pub2 | 2008 |
| 48 | Surgical versus nonsurgical interventions for flail chest | http://dx.doi.org/10.1002/14651858.CD009919.pub2 | 2015 |
| 49 | Surgical versus non‐surgical interventions for displaced intra‐articular calcaneal fractures | http://dx.doi.org/10.1002/14651858.CD008628.pub3 | 2023 |
| 50 | Surgical versus non‐surgical interventions for treating humeral shaft fractures in adults | http://dx.doi.org/10.1002/14651858.CD008832.pub2 | 2012 |
| 51 | Surgical versus non‐surgical treatment for thoracolumbar burst fractures without neurological deficit | http://dx.doi.org/10.1002/14651858.CD005079.pub3 | 2013 |
